# Cerebral myeloid sarcoma (Chloroma): Report of a rare entity

**DOI:** 10.1016/j.ijscr.2025.111233

**Published:** 2025-03-28

**Authors:** Mohamed Aziz Hermassi, Khalil Ghedira, Kais Bouzouita, Alia Zehani, Sofiene Bouali, Khansa Abderrahmen

**Affiliations:** a Department of Neurosurgery, National Institute of Neurology, Tunis, Tunisia; b Department of Pathology, National Institute of Neurology, Tunis, Tunisia

**Keywords:** Acute myeloid leukemia, Chloroma, Granulocytic sarcoma, Brain, Relapse, Case report

## Abstract

**Introduction and importance:**

Myeloid sarcoma is a rare, malignant solid tumor composed of the progenitor cells of the myeloid lineage. Often associated with acute myeloid leukemia. Intracranial localization is very rare and diagnosis is confirmed by immunohistochemistry. The purpose of this case is to highlight the diagnostic and surgical treatment of a pathologically confirmed case of cerebral myeloid sarcoma.

**Case presentation:**

We present the case of a 28-year-old Tunisian man who was diagnosed with AML in 2019, went into remission and was admitted to hospital with headaches and visual disturbances. Cranial MRI revealed an intra-axial parieto-occipital lesion on the left side. The patient underwent a successful surgical removal of the tumor. Immunohistochemistry confirms a cerebral myeloid sarcoma.

**Clinical discussion:**

Myeloid sarcoma is a rare condition. It is mainly found on skin and soft tissue. Intracranial localization is very rare. The clinical presentation of myeloid sarcoma varies depending on the location of the lesion. Histochemistry studies are essential for accurate diagnosis. There is no consensus on the treatment of myeloid sarcoma and different treatment strategies have been used. Surgery plays a key role in alleviating the symptoms of the mass effect, in confirming the diagnosis and in removing the major lesions before initiating systemic therapy.

**Conclusion:**

Cerebral myeloid sarcomas are uncommon. Intracranial mass should be strongly considered in acute myeloid leukemia patients. Conventional chemotherapy for AML is still the primary treatment for myeloid sarcomas, followed by surgery and possibly radiation.

## Highlights

• Cerebral myeloid sarcoma is a rare extramedullary malignant solid tumor composed of myeloid progenitor cells.
• Diagnosis of this extremely rare disease is still challenging and is typically confirmed by immunohistochemical analysis
• We are presenting a very rare case of cerebral myeloid sarcoma in a 28-year-old Tunisian man diagnosed with acute myeloid leukemia.
• The patient underwent successful surgical excision of the lesion.

## 1 Introduction

Myeloid sarcoma (MS), also called chloroma or granulocytic sarcoma, is a rare extramedullary solid tumor composed of progenitors of the myeloid lineage, first described by Burns in 1811 [1]. It is often associated with subtypes of acute myeloid leukemia (AML) such as M2, M4 and M5a [1]. However, it may precede the onset of AML by months to years [1,2]. Any part of the body can be affected, the intracranial region is particularly rare [2]. In 2016, the World Health Organization classified MS as a special subtype of AML [1]. Both sexes are affected equally by MS, and the age of these patients ranges from 2 to 81 years, with a higher incidence in children and adolescents [3].

The clinical presentation of this disease varies depending on the affected brain region [3]. Radiological descriptions of intracranial chloromas are rare in the literature and mainly anecdotal [4]. Currently, MS diagnosis is based primarily on pathologies and immunohistochemistry (IHC) [4]. Conventional chemotherapy for AML is still the primary treatment for MS, as well as surgery and radiation for symptomatic tumors that cause local neurological dysfunction [2,4]. Here we describe a rare case of myeloid sarcoma in a 28-year-old male patient with acute myeloid leukemia. Magnetic resonance imaging (MRI) of the brain revealed a lesion in the left parieto-occipital region. Due to his life-threatening condition, the patient had to undergo emergency surgery. The diagnosis was confirmed by pathology and IHC. The objective of this case report is to highlight the presentation, diagnostic procedure, management strategy and follow-up of this rare disease.

The work has been reported in line with the SCARE criteria and the revised 2023 SCARE guidelines [5].

## 2 Case report

**Fig. 1:**
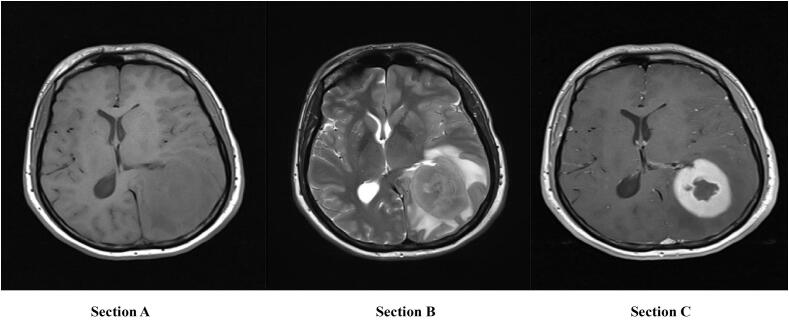
Magnetic resonance imaging (MRI) axial sections with no contrast in T1-weighted imaging (WI) (A); T2-weighted imaging (B) and with contrast in T1 WI (C) showing a left intraaxial parieto-occipital lesion.

In 2019, a 28-year-old Tunisian man was diagnosed with AML M4, underwent standard chemotherapy and was fully recovered within two years. However, in 2021, he was admitted to our facility for two months with headaches and visual disturbances. He reported no seizures or vomiting. No abnormal findings in the examination were noted during the recording period. The laboratory tests were within normal limits. Magnetic resonance imaging (MRI) (Fig. 1) showed a left intra-axial parieto-occipital lesion measuring 6.5 × 8 × 7 cm, isointense at T1-WI and hyper-intense at T2-WI, with homogeneous enhancement and significant surrounding edema. The mass put pressure on the middle structures. Pre-operative differential diagnosis included brain lymphoma, glioma, and hematoma, but none of these were well correlated with clinical, biological, or radiological characteristics.

**Fig. 2:**
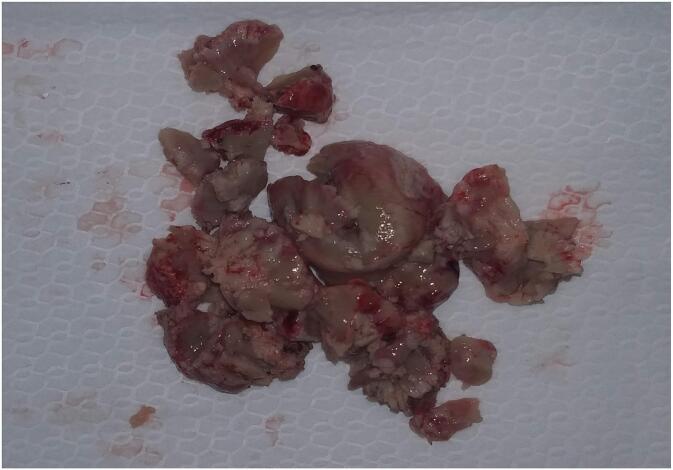
Gross appearance: Cut surface of solid, pink-green, well-defined tumor.

Due to his life-threatening condition, the patient needed emergency surgery. A left parieto-occipital craniotomy was performed and the mass was completely removed. The tumor was homogeneous, solid, green- pink in color and well defined (Fig. 2). It had smooth edges and a rich blood supply, and it was easy to attach to the surrounding cerebral tissue.

**Fig. 3:**
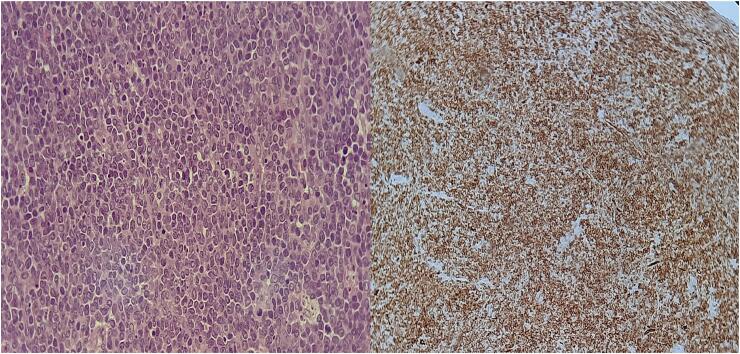
Histological sections show sheets of immature mononuclear cells. Immunohistochemical stains show that immature cells are positive for CD34, MPO and negative for CD19. Morphological findings and immunohistology in the current sample support the diagnosis of myeloid sarcoma.

**Fig. 4:**
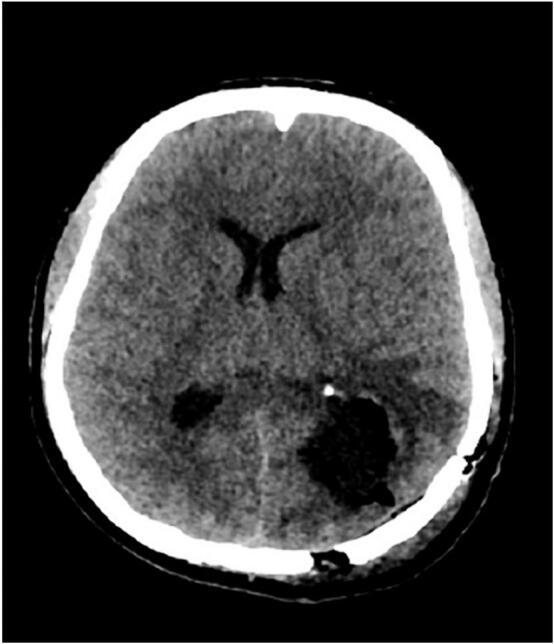
Follow-up computed tomography scan confirming complete removal of the lesion.

The pathological examination of the tumor (Fig. 3) confirmed the diagnosis of MS, and the IHC showed positive markers for MPO and CD34. Immediate surgical follow-up was uneventful and no complications were noted. Subsequent brain computed tomography (CT) (Fig. 4) confirmed complete removal of the mass and satisfactory appearance of surgical site. The case was discussed in a multidisciplinary team meeting that included oncologists, histopathologists, radiologists, and neurosurgeons.

Later, in August 2021, the patient received a single cycle of systemic chemotherapy with high dose cytarabine (6.0 g per m2 on days 1–3) and intermittent intrathecal administration (cytarabine 50 mg, methotrexate 10 mg, dexamethasone 5 mg) 4 times at 0, 4, 8 and 22 days after initiating chemotherapy. Four weeks after the first induction chemotherapy, he received a single combination chemotherapy course (20 mg/m2 on days 1–5, cytarabine 6 g/m2 on days 8–10, mitoxantrone 20 mg/m2 on days 8–10) and intermittent intrathecal injection, followed whole-brain radiotherapy (24 Gy/20 F) in November 2021. A detailed clinical evaluation was performed 6 months after the first treatment to evaluate the effects of the therapy. Cranial MRI showed no recurrence. He remained in full remission for 76 months.

## 3 Discussion

Myeloid sarcoma, also called chloroma or granulocytic sarcoma, rare solid tumor of the white blood cell granulocyte lineage, which is a focal accumulation of leukemic cells [6]. The disease was first described by Burns in 1811 [1]. King renamed it chloroma in 1853 because the typical form is greenish in color because of the high content of MPO [7]. Rappaport renamed it Granulocytic Sarcoma in 1966 when an estimated 30 % of the cells lacked myeloperoxidase [8]. It was later renamed myeloid sarcoma, since some related myeloid leukemias are characterized by a lack of differentiation of granulocytes [6].

The disease is caused by a dense mass of soft tissue destroying the underlying structure of the affected tissue [2,9]. It is usually associated with other myeloid diseases, especially acute myeloid leukemia, as in our case [10]. However, in 35 % of cases, the onset of leukemia may be several months in advance [2,9]. As described in the WHO classification, MS is considered a different presentation and not a subtype of AML [7]. In very rare cases, the disease may be newly diagnosed without history of myeloproliferative or myelodysplastic disease [10]. Its pathogenesis remains unclear, and important theories include hematogenous leukemic cell proliferation, cell attachment to the wall of the blood vessel resulting in local destruction and local proliferation in the skull [9].

MS is a very rare disease, especially in adults [2,10]. It is more common in children and young adults (50–70 %) without gender preference [10]. It is mainly found in the skin, soft tissue and bones, but can also appear in other body parts [11]. Intracranial placement is very rare and the most commonly affected areas are calvaria and orbits [1,3]. Cerebral MS often extends to the meninges or ependymas. However, in rare cases it can invade the brain parenchyma and therefore manifest as intra-axial mass, as in our case [11].

The clinical presentation of cerebral MS varies depending on the site of involvement and often includes headache and visual disturbances due to the mass effect of lesions, although asymptomatic cases have been reported [12]. Specific stain and histochemical studies are required for accurate diagnosis. CT scans and especially MRI are the most widely used imaging techniques for the evaluation of cerebral MS [13]. The mass often appears as a unique extra axial lesion. However, intra-axial lesions such as those described in this case were rarely reported [13,14]. CT often reveals a contorted, isodense or hyper-dense lesion with a slight increase in homogenous contrast and surrounding oedema [15]. MRI is the gold standard and is extremely valuable for surgical strategies. It reveals some aspects that were not clearly visible on the CT scan [15].

Cerebral MS is characteristically observed as hypo-intense or isointense in T1-weighted images and displays heterogeneous isointensity or hyper-intensity on T2-weighted images. Moreover, there is typically a homogeneous increase in signal intensity following gadolinium administration [15,16]. The lesion may present with associated edema and mass effect, as previously elucidated [16]. In AML, these imaging characteristics indicate MS and therefore preclude biopsy or surgery [8]. The lesion may exhibit a morphological resemblance to a meningioma; however, the latter typically demonstrates calcification and hyperostosis rather than osseous destruction [16]. The differential diagnosis encompasses lymphomas, brain abscesses, gliomas, and hematomas. Signal intensity, morphology, and uniform contrast enhancement serve to differentiate these lesions from cerebral MS [8,16].

Histopathological analysis of lesions substantiates the presence of the disease [17]. The surface of freshly excised tumor typically exhibits a green hue as a result of peroxidase-mediated oxidation, whereas approximately 30 % of the surface area remains devoid of this coloration [18]. The tumor cells are characterized by a diffuse distribution, possessing a consistent morphology and exhibiting small to medium dimensions; consequently, in adult patients, they may be misidentified as T-cell lymphoma, diffuse large B-cell lymphoma, or undifferentiated carcinoma [18]. The application of immunohistochemical staining facilitates an accurate diagnostic process [15,18]. Immunohistochemical markers are intricate, and an erroneous interpretation of the results may lead to a misdiagnosis [19].

There is currently no consensus in the literature on the treatment of cerebral MS due to its rarity and different treatment strategies have been used [12,19]. The decision to treat is influenced by factors such as tumor location, time of onset of MS, patient age and status of the disease [16]. Treatment consists of three main categories: systemic or intrathecal chemotherapeutic agents, radiation and surgery [20]. Targeted therapy and immunotherapy represent additional treatment modalities accessible to patients [20].

In our case, the patient had to undergo emergency surgery because of his life-threatening situation. Surgery plays a key role in reducing symptoms of the mass effect, in confirming the diagnosis and in removing major lesions before initiating systemic therapy [11]. The recommended treatment for intracranial MS is combination chemotherapy, similar to that used for AML [11,19]. The combination of 3 days of anthracycline (daunorubicin) and 7 days of cytarabine has been accepted as the standard of care for induction treatment of AML. Inclusion of high-dose of cytarabine in the induction regimens seems essential [20].

Radiotherapy is therefore an alternative treatment that allows local control of the affected area in cases of MS that do not respond to chemotherapy or where rapid relief of vital signs is needed [11,20]. However, it cannot prevent relapses occurring in other places. The prognosis for cerebral MS remains uncertain due to the limited available data [19]. However, it is generally accepted that MS that occurs concomitantly with or as a recurrence of AML is associated with poor prognosis [18,20]. Life expectancy varies according to different factors such as age, state of performance and the affected brain region [20].

## 4 Conclusion

Cerebral myeloid sarcoma is a very rare cranial manifestation of AML. Relapses of AML as isolated intracranial MS are very rare. Patients with AML should maintain a high index of suspicion of extramedullary disease during the follow-up period. The diagnosis of MS depends mainly on pathology and IHC. Conventional chemotherapy protocols for AML are still the primary treatment for cerebral MS, as well as surgery and radiation for symptomatic tumors that cause local neurological dysfunction. The prognosis for this entity is still uncertain and life expectancy depends on many factors.

## Consent

Written informed consent was obtained from the patient for publication and any accompanying images. A copy of the written consent is available for review by the Editor-in-Chief of this journal on request.

## Ethical approval

Our institution (National Institute of Neurology, Tunis) does not require ethical approval for reporting individual cases or case series deemed not to constitute research.

## Guarantor

Mohamed Aziz Hermassi.

## Research registration number

Not applicable.

## Funding

This research did not receive any specific grant from funding agencies in the public, commercial, or not-for-profit sectors.

## Author contribution

Mohamed Aziz Hermassi: The conception and design of the study, drafting the article and revising it critically for important intellectual content, final approval of the version.

Khalil Ghedira: Supervision

Kais Bouzouita: Visualization

Alia Zehani: Data collection

Sofiene Bouali: Review and editing

Khansa Abderrahmen: Validation.

## Registration of research studies

Not applicable.

## Conflict of interest statement

None.
